# *β*-Lapachone Increases Survival of Septic Mice by Regulating Inflammatory and Oxidative Response

**DOI:** 10.1155/2020/8820651

**Published:** 2020-12-12

**Authors:** Ana L. de B. Oliveira, Kely C. Navegantes-Lima, Valter V. S. Monteiro, Lucas B. G. Quadros, Juliana P. de Oliveira, Sávio M. dos Santos, Anna C. A. de A. Pontes, Gilson P. Dorneles, Pedro R. T. Romão, Luiz C. R. Júnior, Alaíde B. de Oliveira, Marta C. Monteiro

**Affiliations:** ^1^Neuroscience and Cellular Biology Post-Graduation Program, Biology Science Institute, Federal University of Pará/UFPA, Belém 66075-900, Brazil; ^2^Center of Research of Inflammatory Diseases, Department of Pharmacology, Ribeirão Preto Medical School, University of São Paulo, Ribeirão Preto 14055230, Brazil; ^3^School of Pharmacy, Health Science Institute, Federal University of Pará/UFPA, Belém 66075-900, Brazil; ^4^Pharmaceutical Sciences Post-Graduation Program, Institute of Health Sciences, Federal University of Pará/UFPA, Belém 66075-900, Brazil; ^5^Laboratory of Cellular and Molecular Immunology, Department of Basic Health Sciences, Federal University of Health Sciences of Porto Alegre, Porto Alegre 90050-170, Brazil; ^6^Department of Pharmaceutical Products, Faculty of Pharmacy, Federal University of Minas Gerais, Belo Horizonte 31270-901, Brazil

## Abstract

Sepsis is characterized by a dysregulated immune response to infection characterized by an early hyperinflammatory and oxidative response followed by a subsequent immunosuppression phase. Although there have been some advances in the treatment of sepsis, mortality rates remain high, urging for the search of new therapies. *β*-Lapachone (*β*-Lap) is a natural compound obtained from *Tabebuia avellanedae* Lorentz ex Griseb. with several pharmacological properties including bactericidal, anti-inflammatory, and antioxidant activity. Thus, the aim of this study was to evaluate the effects of *β*-Lap in a mouse sepsis model. To this, we tested two therapeutic protocols in mice submitted to cecal ligation and puncture- (CLP-) induced sepsis. First, we found that in pretreated animals, *β*-Lap reduced the systemic inflammatory response and improved bacterial clearance and mouse survival. Moreover, *β*-Lap also decreased lipid peroxidation and increased the total antioxidant capacity in the serum and peritoneal cavity of septic animals. In the model of severe sepsis, the posttreatment with *β*-Lap was able to increase the survival of animals and maintain the antioxidant defense function. In conclusion, the *β*-Lap was able to increase the survival of septic animals by a mechanism involving immunomodulatory and antioxidant protective effects.

## 1. Introduction

Sepsis is clinically defined as a life-threatening condition of organ dysfunction caused by a dysregulated immune response to infection [1]. In intensive care units (UCI), it is considered the main cause of death especially in patients with comorbidities [2]. It is estimated to affect more than 30 million people worldwide every year, mainly in low- and middle-income countries, causing 5 million deaths [3]. In sepsis, early activation of both innate and adaptive immune responses leads to an overwhelming inflammatory response, which comprises fever, refractory shock, inadequate resuscitation, and pulmonary or cardiac failure. Meanwhile, mortality at the later period is due to longer immunosuppression with secondary infections resulting in organ injury and/or failure [4]. In the hyperinflammatory phase, immune cells release a great amount of cytokines and reactive oxygen species, causing tissue damage, neutrophil paralysis, severe oxidative stress, and multiple organ failure cellular [5]. In fact, some studies have reported the role of oxidative stress in the pathogenesis and mortality of patients and animals with cecal ligation and puncture- (CLP-) induced polymicrobial sepsis [6, 7].

The drugs currently available for the treatment of sepsis are often unsatisfactory [8]. In the last decades, there has been a renewed interest in natural products as a source of new drugs, including immunomodulatory substances [6, 9]. In this context, we have shown successful strategies for the treatment of CLP-induced septic mice by means of substances/compounds with immunomodulatory and counterregulatory antioxidant and anti-inflammatory actions [6, 7, 9]. In this sense, *β*-Lap is a naturally occurring lipophilic naphthoquinone originally obtained from the heartwood of the lapacho tree (*Tabebuia avellanedae* Lorentz ex Griseb.) native from South America [10]. This quinone is one of the most important lapachol derivatives and has been studied for its interesting chemical and biological properties, such as anticancer [11], trypanocidal [12], bactericidal and fungicidal [13–15], anti-inflammatory [16–18], and antioxidant [19] activities. The mechanisms of *β*-Lap include inhibition in the expression of inducible nitric oxide synthase (iNOS), prostaglandin E2 (PGE2), and proinflammatory cytokines such tumor necrosis factor alpha (TNF-*α*) and interleukin-6 (IL-6) and increase in the expression of anti-inflammatory molecules such as interleukin-10 (IL-10) [16, 20].

Therefore, considering the current problems related to the treatment of sepsis and the urgency for new therapeutic alternatives, we investigated for the first time the effects of prophylactic and therapeutic administration in CLP-induced polymicrobial sepsis in mice.

## 2. Materials and Methods

### 2.1. Ethics Statement

The protocols used in this study were approved by the Institutional Animal Ethics Committee of the Federal University of Pará/UFPA (CEUA, Protocol: 1912080716) and carried out in accordance with recommendations of the Guide for the Care and Use of Laboratory Animals of the Brazilian National Council of Animal Experimentation (http://www.sbcal.org.br/) and the NIH Guidelines for the Care and Use of Laboratory Animals.

### 2.2. Mice

Male Swiss mice (6-8 weeks old) were obtained from the Animal Facility of the UFPA and housed under standard conditions (22°C, 12 h light/12 h dark cycle) with food and water ad libitum in groups of 5 per cage (41 · 34 · 16 cm) in the animal facility of the School of Pharmacy from UFPA, for at least three days before experiments.

### 2.3. Preparation of *β*-Lap

The isolated compound *β*-Lap was kindly donated by Dr. Alaide Braga de Oliveira from the Pharmacy Faculty of the Federal University of Minas Gerais (UFMG). *β*-Lap (3,4-dihydro-2,2-dimethyl-2H-naphthol[1,2-b]pyran-5,6-dione) was synthesized from naturally occurring lapachol derived from T. avellanedae (Bignoneaceae) using sulfuric acid according to a method from Hooker [21]. The solubilization of *β*-Lap was performed in dimethyl sulfoxide (DMSO) at 20 mg/mL stored at −20°C and diluted in saline immediately before use for pretreatment (50 mg/kg) and posttreatment (1 and 5 mg/kg) [10].

### 2.4. Experimental Design and Moderate and Severe CLP Models

The polymicrobial sepsis was induced using the CLP model according to de Souza Gomes et al. Briefly, animals were anesthetized with intraperitoneal injection of 200 *μ*L of ketamine (100 mg/kg) and xylazine (10 mg/kg) solution, a small one-centimeter laparotomy was performed, and the cecum was exposed and then ligated using a 3-0 silk suture [6]. To induce a moderate severity of CLP, the cecum was punctured one single time with a 22-gauge needle, while severe CLP was induced with two perforations using an 18-gauge needle [22]. In protocols, the cecum was gently squeezed to extrude a small amount of fecal content and was left to its original position in the abdominal cavity. Sham-operated mice underwent the same procedure, except for ligation and perforation of the cecum. The abdominal wall was closed, and fluid resuscitation was conducted with subcutaneous injection of 1 mL of saline 0.9% [6].

#### 2.4.1. *β*-Lap in Pretreatment

**Figure 1:**
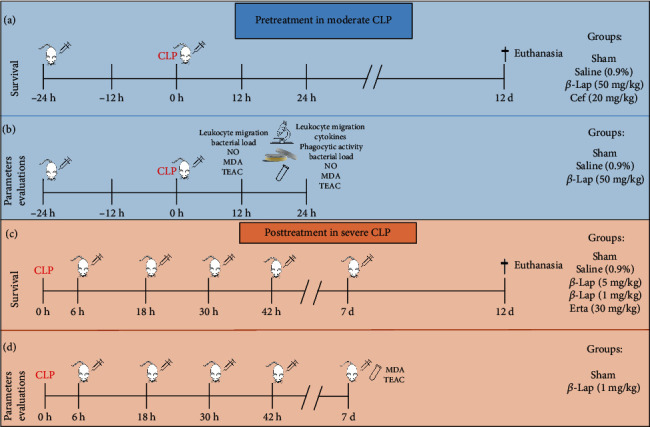
Experimental sets of pretreatment and posttreatment of CLP-induced septic mice with *β*-lapachone. CLP: cecal ligation and puncture; *β*-Lap: *β*-lapachone; CEF: ceftriaxone; NO: nitric oxide; MDA: malondialdehyde; TEAC: Trolox Equivalent Antioxidant Capacity; Erta: ertapenem.

The moderate CLP animals were divided into 4 groups: (1) sham, (2) CLP+saline (0.9%), (3) CLP+*β*-Lap (50 mg/kg), and (4) CLP+ceftriaxone (20 mg/kg; ABL, Brasil). All treatments of the animals were administered intraperitoneally in a volume of 100 *μ*L 24 h before and right after the moderate CLP. In the first experiment, the survival of 28 mice (7 per group) was evaluated. The ceftriaxone group was added as a control, for being an antibiotic that acts against a wide range of pathogens being and used in the prophylaxis of infection and ensuring a longer survival in CLP models [6, 23]. Survival animals were monitored for 12 days (Figure 1(a)). In the second experiment, the anti-inflammatory and antioxidant parameters of 15 mice (5 per group) were evaluated. The animals were then euthanized 12 and 24 hours after surgery, and samples of blood, plasma, and peritoneal fluid lavage were collected and stored at -20°C until analysis (Figure 1(b)).

#### 2.4.2. *β*-Lap in Posttreatment

The severe CLP animals were divided into 5 groups: (1) sham, (2) CLP+saline (0.9%), (3) CLP+*β*-Lap (5 mg/kg), (4) CLP+*β*-Lap (1 mg/kg), and (5) CLP+ertapenem (30 mg/kg; Merck Research Laboratory, Whitehouse Station, NJ, USA) (Figure 1(b)). All treatments of the animals were administered intraperitoneally in a volume of 100 *μ*L beginning 6 hours after severe CLP and 12/12 hours after the first treatment. In the first experiment, the survival of 35 mice (7 per group) was evaluated, and the ertapenem group was added as a control for being an antibiotic with a broad spectrum and used in hospitalized patients with serious infections and increased survival in CLP [24, 25]. The treatment of survival animals remained until the 4th day for group CLP+ertapenem and the 7^th^ day for the other treatments (CLP+saline and CLP+*β*-Lap groups). The survival of the animals was evaluated for 12 days (Figure 1(c)). In the second experiment, the antioxidant parameters of 10 mice (sham and CLP+*β*-Lap 1 mg/kg, 5 per group) were evaluated. The animals were euthanized 7 days after surgery and 6 hours before the last treatment to collect samples of serum and peritoneal fluid lavage and stored at -20°C until analysis (Figure 1(d)).

### 2.5. Survival and Weight Analysis

After CLP induction, the survival of animals (7 mice per group) was monitored each 12 h for 12 consecutive days, and mice were weighted as a parameter of general health. To reduce suffering, mice presenting signs of imminent death (i.e., ataxia, inability to maintain an upright position, tremor, and/or agonal breathing) were euthanized by ketamine/xylazine (>100/10 mg/kg, sc) overdose. The animals that survived for longer than 12 days were euthanized. The survival rate and weight were calculated followed by delineation of survival and weight curve.

### 2.6. Leukocyte Recruitment to the Peritoneal Cavity

After 12 and 24 h of CLP induction in pretreatment groups, the mice were euthanized under ketamine/xylazine, and the peritoneal cavity cells were harvested by washing the cavity with 3 mL of Phosphate-Buffered Saline (PBS) (Sigma-Aldrich, St. Louis, MO, USA) containing ethylenediaminetetraacetic acid (EDTA; NewProv) 1 mM. The recovered volumes were similar in all animals of each experimental group. The total number of leukocytes was determined using a Neubauer chamber, and differential cell counts were carried out on cytocentrifuge (Coulter® ACT; Coulter Corporation, Miami, Florida, USA) slides stained with panotic (Laborclinltda, Pinhais-SP, Brasil). The results are presented as the number of neutrophils and mononuclear cells per cavity.

### 2.7. Phagocytic Activity of Peritoneal Macrophages

The phagocytic capacity of peritoneal macrophages of septic and sham mice was evaluated as previously described. Peritoneal macrophages from sham, CLP-saline, and CLP-*β*-Lap pretreatment groups were collected 24 post moderate sepsis induction and incubated in a 96-well microplate (2 × 10^5^ cells/well) for 40 min at 37°C and 5% carbon dioxide (CO_2_). Two hours later, adherent cells were incubated with 10 *μ*L of neutral-red stained zymosan (Sigma-Aldrich, St. Louis, MO, USA) (1 × 10^8^ particles/mL), and after 30 min, the supernatants were removed and cells fixed with Baker's formol-calcium (4% formaldehyde, 2% sodium chloride, and 1% calcium acetate) for 30 min. Following, the cells were washed two times by centrifugation in PBS (450g for 5 min). After solubilization of neutral-red stain with 0.1 mL of acidified alcohol (10% acetic acid, 40% ethanol in distilled water), the absorbance was measured in a microplate reader at 550 nm [26].

### 2.8. Cytokine Measurement

The levels of TNF-*α* and interleukin-1 beta (IL-1*β*) in serum and peritoneal fluid collected at 24 h post-CLP induction, in the pretreatment groups, were quantified by Enzyme-Linked Immunosorbent Assay (ELISA) using an appropriate commercial kit (R&D Systems, Minneapolis, Canada) according to the manufacturer's instructions. The detection limits of each cytokine were IL-1*β*, 12.5-800 pg/mL with sensitivity of 4.8 pg/mL and TNF-*α*, 10.9-700 pg/mL with sensitivity of 7.21 pg/mL.

### 2.9. Determination of Nitric Oxide (NO) Production

The nitrite (NO_2_) levels were determined colorimetrically using the Griess method according to Romão et al. [27]. After 12 and 24 hours of the CLP induction, nitrite level was determined in 100 *μ*L of samples (serum and lavage peritoneal) from the pretreatment groups incubated with an equal volume of Griess reagent for 10 min at room temperature. The absorbance was measured on a plate scanner (Spectra Max 250; Molecular Devices, Menlo Park, CA, USA) at 550 nm. The nitrite concentration was determined using a standard curve generated using sodium nitrate (NaNO2) and expressed as *μ*Mol/mL.

### 2.10. Bacterial Load Determination

The colony-forming units (CFU) in blood and peritoneal fluid of pretreatment mice were determined 12 and 24 h after CLP induction. An amount of 10 *μ*L of samples was diluted with sterile PBS 1 : 10, and then, 10 *μ*L of each dilution was cultured in Müller Hinton Agar and incubated at 37°C for 24 h. The colonies were counted and expressed in CFU/mL.

### 2.11. Measurement of Malondialdehyde (MDA) Levels

Lipid peroxidation was measured in serum and peritoneal fluid of septic animals from the pretreatment (12 and 24 h after PLC induction) and posttreatment (7 days postsurgery) groups using thiobarbituric acid reactive substance (TBARS; Sigma-Aldrich, St. Louis, MO, USA) assay. An aliquot of 100 *μ*L of the samples was mixed with 0.05 M trichloroacetic acid (TCA; Sigma-Aldrich, St. Louis, MO, USA) and 0.67% thiobarbituric acid (TBA; Sigma-Aldrich, St. Louis, MO) in 2 M sodium sulfate and heated in a water bath at 94°C for 90 min. The chromogen formed was extracted in n-butanol and measured at 535 nm. An MDA standard solution was used to construct a standard curve against which unknown samples were plotted. Results are expressed as malondialdehyde equivalents in nmol/L [28].

### 2.12. Trolox Equivalent Antioxidant Capacity (TEAC) Assay

The total antioxidant capacity (TAC) of serum and peritoneal fluid samples of septic mice from the pretreatment (12 and 24 h after PLC induction) and posttreatment (7 days postsurgery) groups was evaluated by (±)-6-hydroxy-2,5,7,8-tetramethylchromane-2-carboxylic acid (Sigma-Aldrich, St. Louis, MO, USA) equivalent antioxidant capacity assay. In this assay, 2,2′-azino-bis(3-ethylbenzothiazoline-6-sulfonic acid) diammonium salt (ABTS) (Sigma-Aldrich, St. Louis, MO, USA) was incubated with potassium persulphate (Sigma-Aldrich, St. Louis, MO, USA) to produce ABTS^·+^, a green/blue chromophore. The inhibition of ABTS^·+^ formation by antioxidants in the samples was expressed as Trolox equivalents, determined at 740 mm using a calibration curve plotted with different amounts of Trolox (Sigma-Aldrich, St. Louis, MO, USA) [29].

### 2.13. Statistical Analysis

Statistical analyses were performed using the GraphPad Prism 6 software (GraphPad Software Inc., La Jolla, USA). Data are expressed as the mean SD values. Statistically significant differences between groups were determined using analysis of variance (ANOVA) followed by Tukey multiple comparison tests. Survival differences were assessed using the Kaplan–Meier analysis followed by a log-rank test. In all cases, the significance level adopted was 5% (*p* < 0.05).

## 3. Results

### 3.1. *β*-Lap Pretreatment Increased Mononuclear Cell Recruitment and Phagocytosis and Improved Survival of Septic Mice

**Figure 2:**
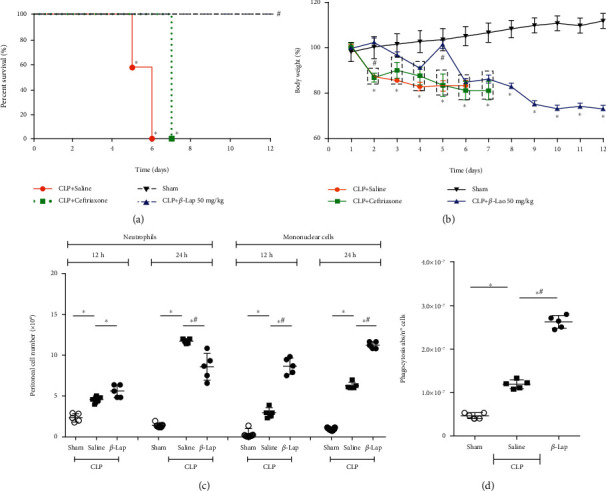
*β*-Lap pretreatment increases septic mouse survival and stimulates mononuclear cell recruitment and phagocytosis. (a) The survival rate of septic mice from saline, *β*-Lap (50 mg/kg), and ceftriaxone (20 mg/kg) groups. (b) Body weight changes. (c) Leukocyte recruitment to infection site at 12 and 24 h post-CLP. (d) The phagocytic capacity of peritoneal cells 24 h after CLP. ^∗^*p* < 0.05 vs. sham; ^#^*p* < 0.05 vs. saline-CLP.

In the moderate CLP model, saline-pretreated septic animals (saline-CLP) survived for 5 days, while 100% of *β*-Lap-pretreated CLP mice (*β*-Lap-CLP) survived up to 12 days after sepsis induction and 60% of ceftriaxone pretreated animals died until the seventh day (Figure 2(a)). The body weight was monitored in order to evaluate the general health condition of animals. As shown in Figure 2(b), all animals presented a drop in body weight after sepsis induction. However, in animals pretreated with *β*-Lap, weight loss was significantly delayed. Regarding leukocyte recruitment towards infectious focus, there was a significant increase in neutrophils and mononuclear cell infiltration into the peritoneal cavity at 12 and 24 h in saline-pretreated septic mice. However, the pretreated with *β*-Lap partially inhibited the neutrophil recruitment at 24 h, while caused a significant increase in mononuclear migration at both 12 and 24 h compared to the saline group (Figure 2(c)). The phagocytic capacity of peritoneal cells of septic animals was elevated at 24 h, and the pretreatment with *β*-Lap potentiated this activity (Figure 2(d)).

### 3.2. *β*-Lap Pretreatment Decreased Inflammatory Cytokines and Bacterial Load

**Figure 3:**
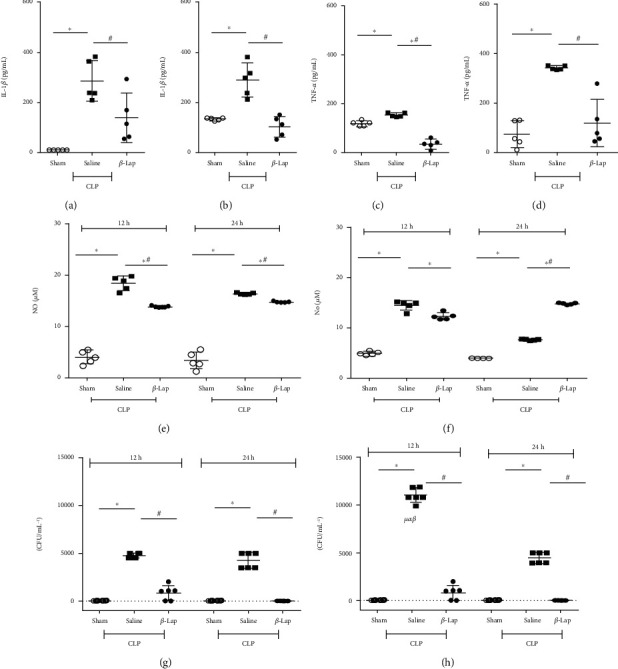
*β*-Lap pretreatment inhibits inflammatory cytokines and bacterial load but does not inhibit nitric oxide in the blood of septic animals. The concentrations of IL-1 *β* in the serum (a) and in peritoneal lavage (b) as well as TNF-*α* in the serum (c) and in peritoneal lavage (d) 24 h after CLP. Nitric oxide levels in serum (e) and in peritoneal lavage (f) 12 h and 24 h after CLP. Bacterial counts (CFUs) in blood (g) and in peritoneal lavage (h). ^∗^*p* < 0.05 vs. sham; ^#^*p* < 0.05 vs. CLP.

Considering the inflammatory pathological response in sepsis, septic mice exhibited higher levels of IL-1*β* and TNF-*α* in serum and peritoneal cavity within 24 h after sepsis induction (Figures 3(a)–3(d)). In contrast, *β*-Lap pretreatment was able to inhibit the production of these cytokines in both sites. Regarding NO, this inflammatory mediator increased in serum and peritoneal cavity of saline-treated septic animals at 12 and 24 h in relation to control animals, while the *β*-Lap-CLP group showed a significant reduction in systemic NO levels with a concomitant increase in peritoneal cavity within 24 h (Figures 3(e) and 3(f)). In addition, the pretreatment of animals with *β*-Lap induced a complete bacterial clearance in blood and peritoneal cavity within 24 h (Figures 3(g) and 3(h)).

### 3.3. *β*-Lap Pretreatment Shows Antioxidant Action with Increased TEAC Levels and Decreased Oxidative Marker in Fluids

**Figure 4:**
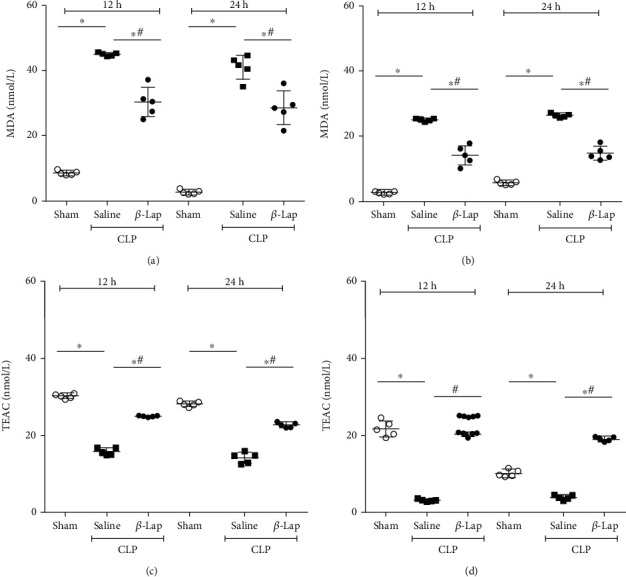
*β*-Lap pretreatment shows antioxidant action by increasing TEAC levels and decreasing MDA in septic animals. MDA levels in the serum (a) and peritoneal fluid (b) at 12 and 24 h post-CLP. TEAC levels in the serum (c) and in peritoneal fluid (d) at 12 and 24 h post-CLP. ^∗^*p* < 0.05 vs. sham; ^#^*p* < 0.05 vs. saline-CLP.

The levels of lipid peroxidation, indicative of oxidative damage, were elevated in saline-CLP mice in serum and peritoneal fluid at 12 and 24 h postinduction. The pretreatment with *β*-Lap was able to reduce MDA levels at both sites (Figures 4(a) and 4(b)). Regarding antioxidant capacity, septic animals pretreated with saline showed a reduction in TEAC levels in the peritoneal cavity and serum at 12 and 24 h, while the CLP-*β*-Lap group showed high levels of total antioxidant capacity (Figures 4(c) and 4(d)).

Table 1 shows the MDA/TEAC ratios in peritoneal fluid and serum at 12 and 24 h post-CLP, confirming that saline-pretreated septic animals had high oxidative status and that the pretreatment with *β*-Lap lead to a significant reduction in this oxidative profile.

### 3.4. *β*-Lap Posttreatment Improved the Survival Rate of Severe Septic Mice and Restored Basal MDA and TEAC Levels

**Figure 5:**
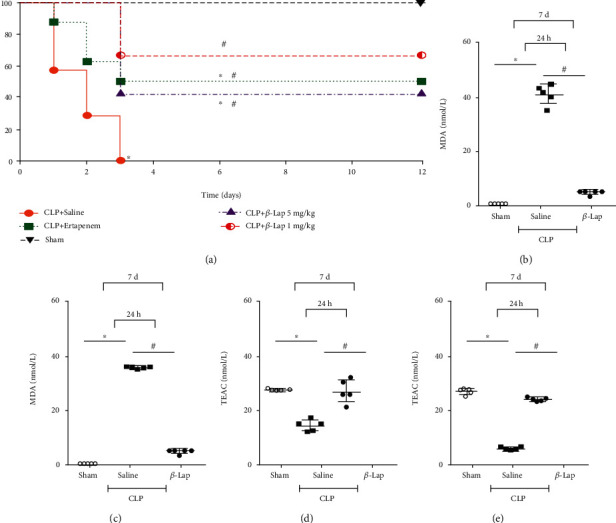
*β*-Lap posttreatment improves survival of severe septic mice and restores basal MDA and TEAC levels. (a) The survival rate of septic mice from saline, *β*-Lap (1 mg/kg/5 mg/kg), and ertapenem (30 mg/kg) groups. The MDA level rate of septic mice from saline (24 h post-CLP) and *β*-Lap (1 mg/kg), ertapenem (30 mg/kg) groups 7 days after surgery in the serum (b) and peritoneal lavage (c) TEAC levels of septic mice from saline (24 h post-CLP) and *β*-Lap (1 mg/kg), ertapenem (30 mg/kg) groups 7 days after surgery in the serum (d) and in peritoneal lavage (e) 7 days after surgery. ^∗^*p* < 0.05 vs. sham; ^#^*p* < 0.05 vs. CLP.

All severe septic mice posttreated with saline died within 3 days. However, the posttreatment with *β*-Lap was able to increase the survival of animals up to 12 days, reaching 60 and 50% survival rate at doses of 1 and 5 mg/kg, respectively. On the other hand, ertapenem also increased the survival of animals with severe sepsis, reaching 54% survival on day 12 after sepsis (Figure 5(a)). Moreover, we found that septic animals treated with *β*-Lap (1 mg/kg) showed decreased levels of MDA in serum and peritoneal fluid at the 7^th^ day of sepsis compared to the moderate CLP group at 24 h postinduction (Figures 5(b) and 5(c)). Furthermore, *β*-Lap-treated severe septic animals also presented higher TEAC values compared to animals with moderate sepsis saline-treated at 24 h of infection (Figures 5(d) and 5(e)).

Therefore, Table 2 shows that there is no significant difference in oxidative status between sham and *β*-Lap groups.

## 4. Discussion

In the present study, we showed for the first time the protective effect of *β*-Lap in an experimental sepsis model. The prophylactic treatment with *β*-Lap improved the survival of mice in a model of moderate sepsis, regulating neutrophils and mononuclear cell infiltration into the peritoneal cavity and improving phagocytic capacity. *β*-Lap showed immunomodulatory actions reducing proinflammatory cytokines (IL-1*β* and TNF-*α*) and NO production in the serum, with a concomitant increase in NO production and bacteria killing in the peritoneal cavity. This protective effect was accompanied by a reduction in lipid peroxidation and an augment in the antioxidant status. On the other hand, the posttreatment with *β*-Lap in the severe sepsis model was able to improve the survival rate of septic animals by a mechanism that involves the maintenance of antioxidant defense.

*β*-Lap is a bioreductive prodrug that undergoes a reduction of two electrons via nicotinamide adenine dinucleotide phosphate (NAD (P) H) quinone oxidoreductase 1 (NQO1). The reduced form of *β*-Lap is unstable and therefore is quickly oxidized to the original form of *β*-Lap, creating a futile cycle between the oxidized and reduced forms with the production of reactive oxygen species (ROS), such as superoxide anion (O_2_^-·^) and hydrogen peroxide (H_2_O_2_) [30]. This compound can cause toxicity due to the subsequent depletion of nicotinamide adenine dinucleotide (NADH) or NAD (P) H, catalyzed by NQO1, together with the signal transduction of the oxidative stress trigger by cell death [31]. In fact, the compound has been reported as a promising antitumor candidate drug, since the cytotoxicity of *β*-Lap can selectively kill cancer [32].

The selectivity of this compound for tumors is associated with the fact that cancer cells generally have higher NQO1 compared to normal cells [32]. However, specificity and selectivity for NQO1 depend on cytotoxicity that can be achieved only within a relatively short dose range and within a short period of time [33]. The chronic exposure of mice to the compound can produce unpredictable toxic effects in high doses, time, and frequency of exposure [34]. In order to prevent possible toxicity, given that there is a higher frequency of administration and for a longer time considering a possible clinical usage, we sought to evaluate if lower doses would be effective in a longer period of time in the posttreatment of the CLP. Thus, we evaluated the response of prophylactic treatment of *β*-Lap in the model of moderate sepsis with a dose of 50 mg/kg and the posttreatment effect using a model of severe sepsis and a lower dose of *β*-Lap (1 mg/kg and 5 mg/kg).

In sepsis, phagocytic cells are recruited to the site of infection in the initial phase of infection. Neutrophils are the first cells to be recruited and exert potent antimicrobial activity through ROS, reactive nitrogen species (RNS), or granule proteins, which act in support to the microbial killing in phagosome [35, 36]. In addition to phagocytosis, neutrophils act through the formation of networks of extracellular traps (NETs) composed of deoxyribonucleic acid (DNA), histones, antimicrobial proteins, and oxidizing enzymes that are released to trap microbes [37]. The participation of reactive species such as NO is essential for bacteria killing, and its absence can lead to uncontrolled infections, despite the improvement of cell migration to the infectious focus [37]. In this model of moderate sepsis, prophylactic treatment with *β*-Lap increased in 12 h after CLP neutrophil recruitment to the peritoneum, the focus of infection in CLP sepsis and subsequent bacterial growth is better controlled, and survival significantly improved. On the other hand, in our model of severe sepsis and posttreatment with *β*-Lap at a dose of 1 mg/kg, no change inflammatory parameters such as cytokines, mononuclear recruitment, and phagocytosis (unpublished data) were compared to saline-posttreated sepsis animals.

Although essentials for eradicating a wide range of pathogens in the early stages of infection, neutrophil recruitment and overactivation can induce inflammation and deleterious immune responses [5]. The emigrating of neutrophils from the bloodstream can cause tissue and endothelial damage, inducing thrombosis and edema by releasing oxidant compounds, proteases, and cytokines [38]. We found that 24 h after CLP, the prophylactic treatment with *β*-Lap was able to reduce neutrophil recruitment. The transgenic zebrafish model was also observed that the *β*-Lap compound caused reduced recruitment of neutrophils to the injury site, without affecting the resolution of inflammation [39]. Moreover, it was demonstrated that the topical treatment of *Leishmania major*-infected BALB/c mice with *β*-Lap loaded in lecithin-chitosan nanoparticles downregulated IL-1*β* and cyclooxygenase (COX-2) in the draining lymph nodes and reduced the cutaneous lesion size and neutrophil infiltration, without affecting parasites burdens [40].

During sepsis progression, neutrophil can decrease phagocytosis, ROS production, and granular activity [37]. In this study, prophylactic treatment with *β*-Lap maintained the neutrophil activity observed by the increase in phagocytic capacity and NO production in the inflammatory site 24 h after CLP, which may have contributed to the bacterial control and survival of animals. Moreover, studies reported that *β*-Lap induced antibacterial activity through the generation of O_2_^-·^ and H_2_O_2_ [13, 14]. In addition, our study showed that *β*-Lap pretreatment modulates mononuclear cells into the peritoneal cavity increasing the recruitment of these cells. The recruiting of mononuclear cells of the peritoneal cavity by *β*-Lap apparently consists in macrophages, cells essential for the active process for the control of sepsis [41]. Collaborating with our data, studies conducted in the mouse model of wound healing found that the treatment with *β*-Lap for 22 days promoted macrophage proliferation and accelerated cicatrization [42]. Macrophages comprise a heterogeneous population of cells that can become polarized into distinct subpopulations displaying either a classically activated (M1) or alternatively activated (M2) profile [43]. While M1 macrophages are characterized by producing proinflammatory cytokines (TNF-*α*, IL-1*β*, IL-6, etc.), the profile M2 is involved in tissue repair and in the regulation of the excessive inflammatory response [43]. Mehta et al. evaluated in baboons with experimental peritonitis induced by an E. coli-laden fibrin clot the immunoinflammatory sequelae in sepsis and septic shock and observed that monocytes from baboons that died largely displayed the characteristics of classically activated M1 macrophages [44].

In this study, the prophylactic treatment with *β*-Lap mitigates the excessive release of TNF-*α*, IL-1*β* in serum, and peritoneal cavity of animals 24 hours after CLP, suggesting a polarization to M2 phenotype, which is associated with the downregulation of nuclear factor kappa B (NF-*κ*B) pathway that in sepsis promotes excessive release of IL-1*β* and TNF*α*, inflammatory cytokines central to the pathophysiology and partly involved in the pathogenesis of multiple organ failure [45]. In fact, previous studies *in vitro* and *in vivo* have demonstrated that *β*-Lap is able to inhibit the phosphorylation of protein tyrosine and NF-*κ*B activation [16, 46]. Therefore, blockage in the production of these proinflammatory cytokines through inhibition of NF-*κ*B may prevent the excessive inflammatory reaction during sepsis [47].

In our model, we observed the reduction of NO in the serum of *β*-Lap-pretreated animals. Endothelial-derived NO dilates blood vessels by relaxing vascular smooth muscle and maintains microvascular homeostasis [48]. Although NO is important for the regulation of blood pressure, its excessive augmentation can lead to severe hypotension and shock due to vascular instability and myocardial depression [49]. Thus, the anti-inflammatory and protective effect of *β*-Lap can be in part due to the inhibiting of systemic NO. In agreement, it was reported that *in vitro*, *β*-Lap inhibited the expression of iNOS, proinflammatory cytokines, and matrix metalloproteinases 3, 8, and 9 at messenger ribonucleic acid (mRNA) and protein levels in lipopolysaccharide- (LPS-) stimulated microglia, by inhibiting mitogen-activated protein kinase (MAPKs), via phosphatidylinositol 3 kinase/protein kinase B (PI3K/AKT), and nuclear factor kappa B/activator protein 1 (NF-*κ*B/AP-1) signaling pathways in LPS-stimulated microglia [16]. Furthermore, it also inhibited the neuroinflammation induced by systemic administration of LPS in an inflammation mouse model [16].

Another feature of sepsis is the excessive production of ROS associated with inflammation that leads to a condition of oxidative stress [32]. This oxidative imbalance promotes macromolecule damage, including lipid peroxidation and oxidation of proteins and DNA [49]. High serum MDA levels have been associated with severity and mortality in septic patients [50]. In the present study, the levels of MDA were reduced in serum and peritoneal fluid of animals pretreated and posttreated with *β*-Lap. Additionally, *β*-Lap also increased the antioxidant capacity of these treated animals. In agreement, *β*-Lap has been reported to positively regulate the signaling pathway of the antioxidant response element nuclear factor erythroid-2-related factor 2 (Nrf2)/antioxidant response element (ARE), an important mechanism in cellular defense against oxidative or electrophilic stress [16, 19, 51]. In LPS-stimulated BV2 microglial cells, it was observed that *β*-Lap increased Nrf2 binding to ARE and induced phase II antioxidant enzymes [16]. In doxorubicin-induced cardiotoxicity, the *β*-Lap elevated the nuclear accumulation of Nrf2 and upregulated the protein levels of glutathione in the heart, as well as the activity of cardiac superoxide dismutase (SOD), glutathione peroxidase (GPX), and catalase (CAT), improving the cardiac function of treated mice [51]. In patients with sepsis, the decreased antioxidant capacity is associated with death [52].

In our study, *β*-lapachone pretreatment at the dose (50 mg/kg) was able to prevent tissue damage caused by an exacerbated immune response and oxidative stress induced by sepsis. We also found that *β*-Lap posttreatment in lower doses, mainly 1 mg/kg, increases the survival of septic animals and reduced the tissue damage by antioxidant mechanisms but did not alter the immune response of septic animals. Thus, these data, together, suggest that *β*-lapachone has both prophylactic and therapeutic effects, because even in low doses and in posttreatment after sepsis, this compound was able to prevent animal death and tissue damage caused by the immune response to infection and oxidative processes.

## 5. Conclusions

**Figure 6:**
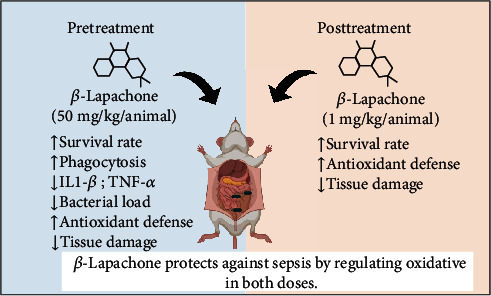
*β*-Lap enhanced survival and reduced organ damage and increases antioxidant defense in the CLP sepsis model in both doses (50 mg/kg; 1 mg/kg).

In summary, taken together, our results reinforce, mainly the antioxidant capacity of *β*-Lap, and demonstrate for the first time its prophylactic and therapeutic effects in CLP-induced sepsis as summarized in Figure 6. *β*-Lap was able to increase the survival of septic animals by a mechanism involving immunomodulatory (increased mononuclear recruitment, phagocytosis, NO production, and bacterial killing), but mainly by an antioxidant mechanism. Further studies are needed to better elucidate the therapeutic mechanisms and ensure the safety of their clinical use.

## Figures and Tables

**Table 1:** Ratios between MDA levels and antioxidant (TEAC) factor of groups 12 and 24 hours after surgery.

	MDA/TEAC mean ± SD					
	Serum			Peritoneal lavage		
Time	Sham	Saline+CLP	*β*-Lap+CLP	Sham	Saline+CLP	*β*-Lap+CLP
12 h	0.18 ± 0.10	2.51 ± 0.62^∗^	1.16 ± 0.43^∗#^	0.12 ± 0.01	8.00 ± 1.09^∗^	0.68 ± 0.22^∗#^
24 h	0.09 ± 0.04	2.86 ± 0.95^∗^	1.26 ± 0.50^∗#^	0.41 ± 0.22	7.19 ± 0.12^∗^	0.76 ± 0.15^∗#^

^∗^ *p* < 0.05 in relation to the sham group; ^#^*p* < 0.05 in relation to the saline-CLP group.

**Table 2:** Ratios between MDA levels and antioxidant (TEAC) factor of groups 7 days after surgery.

	MDA/TEAC mean ± SD					
	Serum			Peritoneal lavage		
Time	Sham	Saline+CLP^&^	*β*-Lap+CLP	Sham	Saline+CLP^&^	*β*-Lap+CLP
7 d	0.02 ± 0.01	2.86 ± 0.95^∗^	0.11 ± 0.04^#^	0.01 ± 0.01	7.19 ± 0.12^∗^	0.05 ± 0.02^#^

^&^Saline-CLP group 24 h; ^∗^*p* < 0.05 in relation to the sham group; ^#^*p* < 0.05 in relation to the saline-CLP group.

## Data Availability

All data generated or analyzed during this study are included in the article.
